# Structural Prediction and Mutational Analysis of the *Gifsy-1 *Xis Protein

**DOI:** 10.1186/1471-2180-8-199

**Published:** 2008-11-17

**Authors:** Asa Flanigan, Jeffrey Gardner

**Affiliations:** 1 Department of Microbiology, University of Illinois in Urbana-Champaign, Urbana, Illinois, USA; 2 College of Medicine, University of Illinois in Urbana-Champaign, Urbana, Illinois, USA

## Abstract

**Background:**

The *Gifsy-1 *phage integrates into the *Salmonella *Typhimurium chromosome via an integrase mediated, site-specific recombination mechanism. Excision of the *Gifsy-1 *phage requires three proteins, the *Gifsy-1 *integrase (Int), the *Gifsy-1 *excisionase (Xis) protein, and host encoded Integration Host Factor (IHF). The *Gifsy-1 xis *gene encodes the 94-residue *Gifsy-1 *excisionase protein that has a molecular weight of 11.2 kDa and a pI of 10.2. Electrophoretic Mobility Shift Assays (EMSA) suggested at least one region of the protein is responsible for protein-DNA interactions with a tripartite DNA binding site composed of three direct imperfect repeats.

**Results:**

Here we have undertaken experiments to dissect and model the structural motifs of *Gifsy-1 *Xis necessary for its observed DNA binding activity. Diethyl sulfate mutagenesis (DES) and mutagenic PCR techniques were used to generate *Gifsy-1 xis *mutants. Mutant Xis proteins that lacked activity in vivo were purified and tested by EMSA for binding to the *Gifsy-1 *Xis *attP *attachment site. Results from mutagenesis experiments and EMSA were compared to results of structural predictions and sequence analyses.

**Conclusion:**

Sequence comparisons revealed evidence for three distinct structural motifs in the *Gifsy-1 *Xis protein. Multiple sequence alignments revealed unexpected homologies between the *Gifsy-1 *Xis protein and two distinct subsets of polynucleotide binding proteins. Our data may suggest a role for the *Gifsy-1 *Xis in the regulation of the *Gifsy-1 *phage excision beyond that of DNA binding and possible interactions with the *Gifsy-1 *Int protein.

## Background

Temperate phages are capable of following alternative life styles [1]. Under appropriate conditions, they grow lytically to form progeny phage particles and lyse the host cell. Alternatively, they can form lysogens where they either integrate their chromosomes by site-specific recombination or exist as autonomous extra chromosomal plasmids. Because temperate phages are able to move between hosts, they serve as vectors for horizontal gene transfer in bacteria. Temperate bacteriophages often carry accessory genes that are not necessary for growth or survival of the phage. For example phage *Gifsy-1 *whose host is *Salmonella *Typhimurium, carries genes that encode pathogenicity factors [2-6]. The *Gifsy-1 gipA *gene product contributes to the survival of *Salmonella *Typhimurium in the Peyer's patches of the mouse intestine and, along with other pathogenicity factors encoded on the related *Gifsy-2 *phage, contributes to the ability of the bacterial pathogen to survive within a host organism and cause disease [7].

*Gifsy-1 *integrates into and excises from the host chromosome by site-specific recombination. Integration of *Gifsy-1 *into the host chromosome requires the phage encoded Integrase (Int) that catalyzes recombination between a 14 base pair core attachment site in the phage chromosome (*attP*) and an identical sequence in the host chromosome (*attB*). Integration also requires the *Salmonella *Typhimurium encoded integration host factor (IHF) that likely acts with Int to form a nucleoprotein structure called the intasome. This higher order complex facilitates synapsis and the recombination reaction.

Deletion analysis showed that excision of *Gifsy-1 *requires Int and a protein, called Xis, encoded by the phage *xis *gene [8]. The *Gifsy-1 *Xis protein is a typical recombination directionality factor (RDF) because it is a small (94 residue) and highly basic (pI = 10.2) protein [2,3,5,9]. Electrophoretic mobility shift assay (EMSA) and footprinting analyses identified a tripartite Xis binding site in *attP *DNA. This initial analysis of the protein-DNA interactions also demonstrated that tight, specific binding of the *Gifsy-1 *Xis protein to DNA involves sequential and cooperative interactions of three Xis protomers to the minimal *attP *binding site. These observations suggested that protein-protein interactions might occur between *Gifsy-1 *Xis monomers similar to results reported for the lambda Xis protein [9-11].

While the *Gifsy-1 *phage carries genes that affect the pathogenicity of *Salmonella *Typhimurium, the site-specific recombination system has not been characterized. To date only the DNA binding sites of the *Gifsy-1 *phage Xis protein have been characterized. Here we begin a mutational and structural analysis of the *Gifsy-1 *Xis protein to provide more information on the molecular mechanism of *Gifsy-1 *excision.

## Results and discussion

### Multiple Sequence Alignment (MSA) of the *Gifsy-1 Xis *protein with RDFs and Transcription Factors

**Figure 1:**
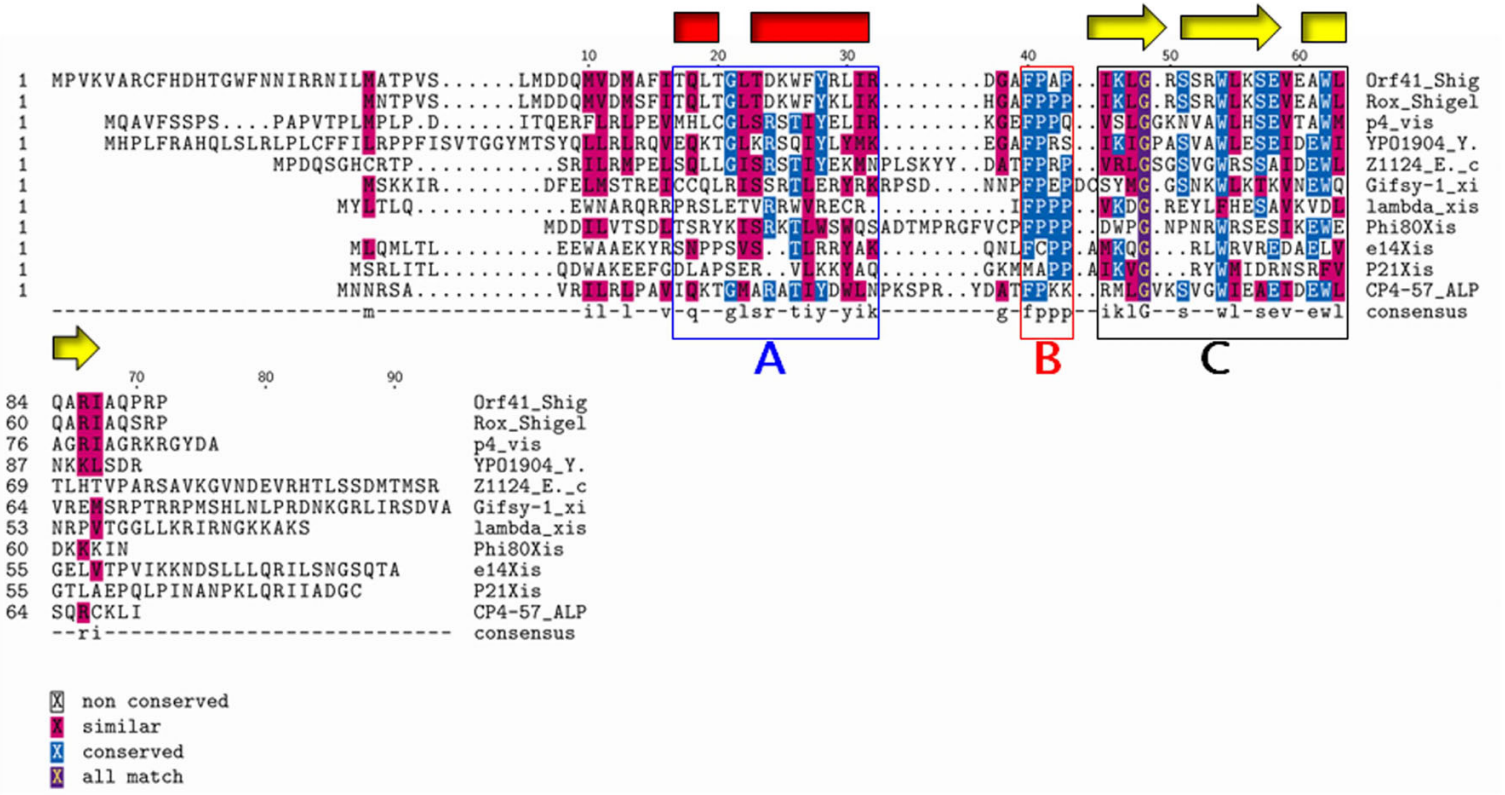
**CustalW analysis of *Gifsy-1 *Xis and proteins containing proline loops.** A residue ruler is indicated along the top of the figure and is numbered according to the *Gifsy-1 *Xis sequence. The residue number for individual proteins is indicated on the left-hand side of the figure. Residues aligned with the *Gifsy-1 *Xis H-T-H motif are included in the blue box A. Residues aligned with the *Gifsy-1 *Xis FPPP loop are included in the red box B. Residues aligned with the *Gifsy-1 *Xis β-sheet are included in the black box C. The secondary structures assigned to the *Gifsy-1 *Xis protein are aligned with the sequences along the top of the diagram. Red rectangles indicate α-helix sequences and yellow arrows indicate β-sheet structures, respectively. The proteins listed from top to bottom are as follows: *Shigella *flexneri Orf41, *Shigella *flexineri Rox, P4 Vis, *Yersinia *Pestis YPO1904, *E*. coli Z112, *Salmonella *Typhimurium *Gifsy-1 *Xis, phage lambda Xis, *E*. coli phage Phi80 Xis, phage e14 Xis, phage P21 Xis, CP4-57 ALPA.

NMR and crystallization of the lambda Xis protein revealed the presence of a short proline loop that connects the N-terminal α-helix region to a β-sheet "wing" structure [11,12]. The Clustal W program [13,14] was used to perform multiple sequence alignment (MSA) of the *Gifsy-1 *Xis protein with predicted and characterized RDF (recombination directionality factor) proteins and certain transcription factors [15]. This study indicated that several other RDF proteins and transcription factors also contain proline loop sequences. Our results also indicated that *Gifsy-1 *Xis shows significant homology to these proteins in a short proline loop region (*Gifsy-1 *Xis residues P39 to P43) (Figure 1). Thus, our results suggest that this proline loop is a conserved motif in a set of transcription factors and RDF proteins.

*Gifsy-1 *Xis possesses limited homology to these proteins in two other regions. The aligned proteins, *Shigella *flexneri Orf41, *Shigella *flexineri Rox, P4 Vis, *Yersinia *Pestis YPO1904, *E*. coli Z112, *Salmonella *Typhimurium *Gifsy-1 *Xis, phage Phi80 Xis, CP4-57 ALPA, share limited similarity in the putative helix turn helix (H-T-H) motif (*Gifsy-1 *Xis residues C18 to K32). An additional region of homology corresponds to the three strand β-sheet of the *Gifsy-1 *Xis protein (residues S46 to S68) that are C-terminal to the proline linker region. *Salmonella *Typhimurium *Gifsy-1 *Xis, *E*. coli phage Phi80 Xis, phage e14 Xis, phage P21 Xis, CP4-57 ALPA, proteins possess several conserved residues in this region. Studies with the *Gifsy-1 *Xis protein suggests that all three regions, the N-terminal H-T-H, the proline loop, and the C-terminal β-sheet, play a role in DNA binding. Our alignment results may indicate that these several proteins may use three regions for binding to DNA.

**Figure 2:**
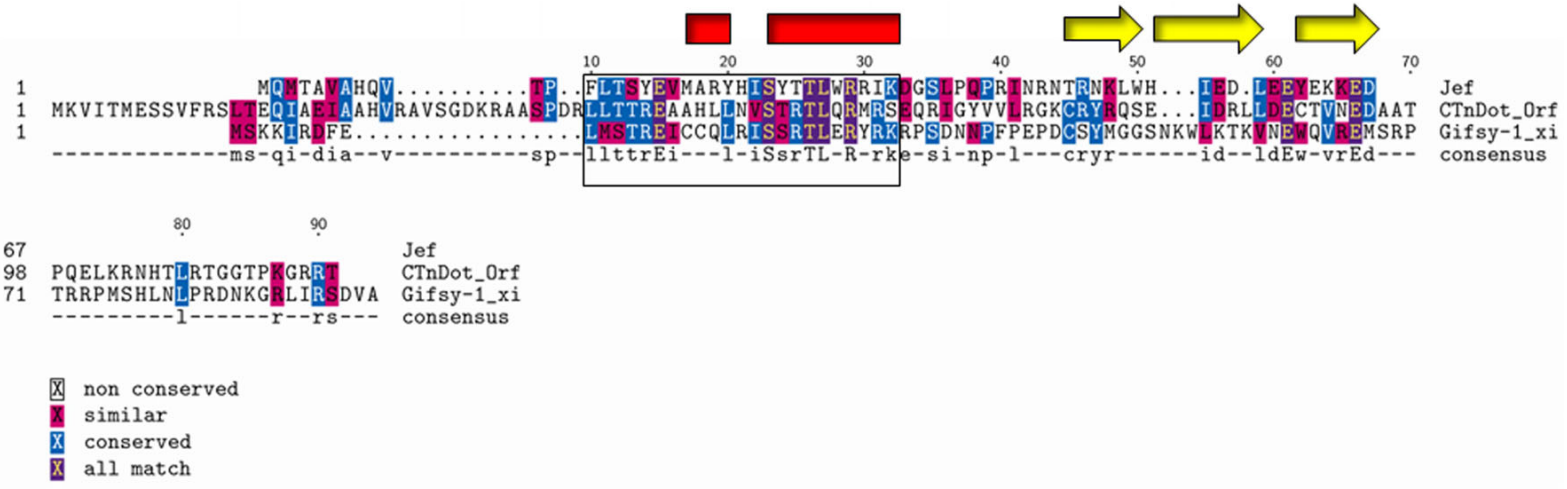
**Clustal W alignment of the R391 Jef, CTnDOT Orf2c, and *Gifsy-1 *Xis. **A residue ruler is indicated along the top of the figure. The ruler corresponds to the residue number of the *Gifsy-1 *Xis protein. The residue number for individual proteins is indicated on the left-hand side of the figure. The boxed sequences indicate the region of homology shared by the three proteins.

We also performed multiple sequence alignment studies of the *Gifsy-1 *Xis protein with the CTnDOT Orf2c protein [9] and the RDF of the conjugative element R391 that is identical to the characterized Xis of the R391 related *Vibrio Cholera *phage SXT [16,17], (Figure 2). All three proteins share homology in an N-terminal region corresponding to residues 40 to 62 of CTnDOT Orf2c, residues 10 to 32 of *Gifsy-1 *Xis, and residues 13 to 35 of the R391 Jef protein. Each short sequence of homology is predicted to form a H-T-H motif with a probability of 100% for CTnDOT Orf2c, 25% for *Gifsy-1 *Xis, and 50% for the Jef protein [18].

These several alignment studies suggest that the regions of the *Gifsy-1 *Xis protein are related to two distinct groups of proteins. The first group contains a wide variety of DNA binding proteins in which the homology is located mostly in the proline loop and a putative β-sheet. The second group includes the RDFs of conjugative elements. The sequence conservation in this group is located in the putative N-terminal H-T-H motifs of the RDFs. While members of the two groups possess limited homology to the *Gifsy-1 *Xis protein, the groups do not show significant homology to each other.

### Structure Prediction and Motif Analysis of *Gifsy-1 *Xis

**Figure 3:**
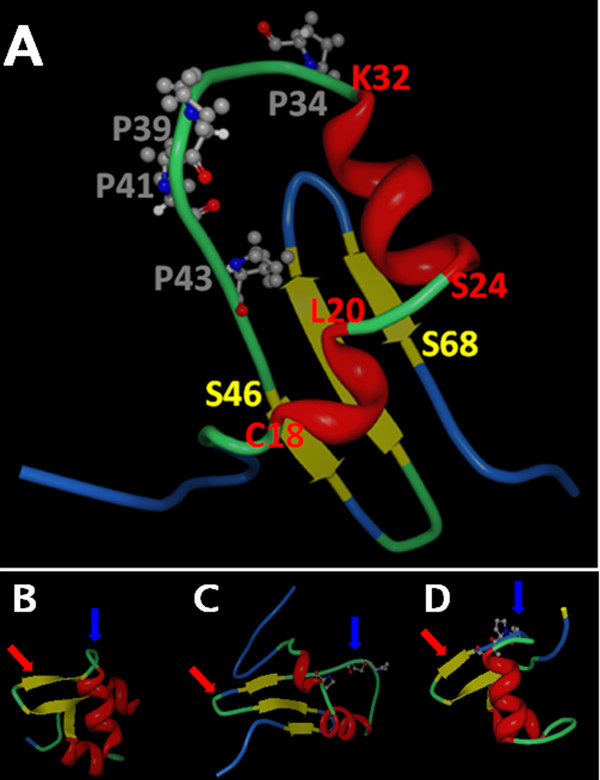
**A. Robetta generated model of the *Gifsy-1 *Xis residues 1 to 72.** Red ribbons indicate α-helices. Yellow ribbons indicate β-sheets. The numbers next to the model delimit predicted resolved motifs and are colored according to the corresponding ribbon structure: Helix 1 = C18 to L20; Helix 2 = S24 to K32; β-sheet structure = S46 to S68. Proline residues involved in the "Proline loop" are indicated by ball and stick models (P39, P41, P43) and are indicated by grey numbers. B-D. Robetta 3-dimensional Structure prediction of *Gifsy-1 *Xis compared to empirical structure determinations. B. Tn916 Xis NMR structure [34]; C. *Gifsy-1 *Xis de novo Robetta prediction; D. lambda Xis [11]. Red arrows indicate "wing" structures. Blue arrows indicate positioning loops. Proline loop motifs are indicated by rendered ball-and-stick atom structures.

The *Gifsy-1 *Xis protein, like the lambda and Tn916 Xis proteins, is a small basic DNA binding protein. Previous DNA binding studies suggest that *Gifsy-1 *Xis interacts with DNA in a similar manner to the lambda Xis protein. These similarities led us to use a modeling technique to determine if *Gifsy-1 *Xis protein could fold into a structure that is comparable to the structure of the two similar proteins, the lambda Xis protein, and the Tn916 Xis proteins, for which structural data exists. The Robetta program for 3-dimensional protein structure prediction [19] was used to generate a tertiary folding prediction for the *Gifsy-1 *Xis protein. A model with a reasonable Z-score (> 6.0) was generated by Robetta (Figure 3A). The model folded the first 72 N-terminal residues of the 94 residue *Gifsy-1 *Xis. Thus, our model includes two N-terminal α-helixes (residues C18 to L20 and S24 to K32) followed by a loop containing the proline-rich loop (residues R33 to C45). The loop connects the second helix to a 3-strand β-sheet that spans residues S46 to S68. Both *Gifsy-1 *Xis and lambda Xis contain an N-terminal H-T-H motif linked to a β-sheet by an intervening proline-rich loop. The overall structural arrangement is similar to the solved NMR and crystal structures of the lambda Xis (residues 1 to 55) and the Tn916 Xis proteins (Figure 3B, 3C). The Robetta software did not resolve the remainder of the protein from residues 73 to 94.

### Limited proteolysis of the *Gifsy-1 *Xis protein

**Figure 4:**
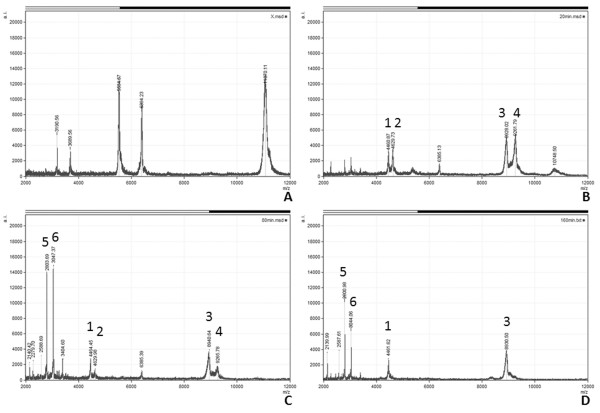
**Time course of *Gifsy-1 *Xis digested with proteinase K.** Lane 1: Molecular mass marker. Lane 2: Undigested *Gifsy-1 *Xis. Lanes 3 to 6 contain *Gifsy-1 *Xis digested with proteinase K (proteinase to protein molar ratio of 1:8). Lane 3: 5 minutes. Lane 4: 10 minutes. Lane 5: 20 minutes. Lane 6: 40 minutes.

Limited proteolysis was performed using proteinase K to identify regions of the protein resistant to proteolysis. The cleavage properties of proteinase K predict that there are multiple potential sites of cleavage across the entire length of the *Gifsy-1 *Xis protein [20-22]. It is predicted that unstructured loops will be digested more rapidly by the broad specificity proteinase K than structured domains and motifs. Thus, fragments of *Gifsy-1 *Xis that remain after extended periods of proteolysis would result from the presence of compact structural motifs [23]. MALDI-TOF analysis of undigested *Gifsy-1 *Xis protein resulted in a peak of approximately 11.1 kDa (Figure 4A). This mass matches the predicted mass of *Gifsy-1 *Xis within the margin of error of the MALDI-TOF apparatus used in these studies. Two fragments of 5.5 kDa and 6.4 kDa were generated by the ionization of the protein. These fragments were labile to proteolysis and were not observed in significant amounts in spectra produced by the proteolysis of the *Gifsy-1 *Xis protein with proteinase K.

**Figure 5:**
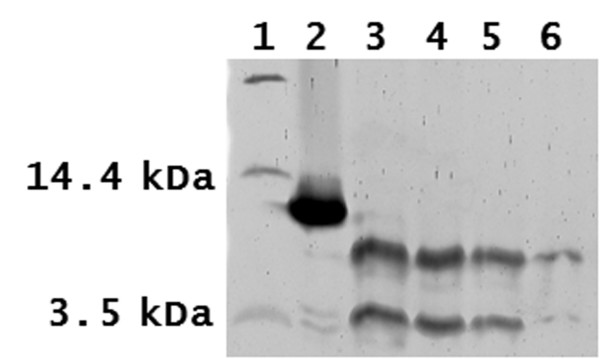
**MALDI-TOF analysis of *Gifsy-1 *Xis digested with proteinase K. **Panel A contains a spectrum of undigested *Gifsy-1 *Xis. Panel B contains a spectrum of 20 minutes post addition of proteinase K. Panel C contains a spectrum 80 minutes post addition of proteinase K. Panel D contains a spectrum 160 minutes post addition of proteinase K. Peak identities are as follows. Peak 1: Residues 3–35, KKIRDFELMSTREICCQ LRISSRTLERYRKRPS; Peak 2: Residues 3–36, KKIRDFELMSTREICCQLRISSRTLERYRKRPSD; Peak 3: Residues 6–73, RDFELMSTREICCQLRISSR TLERYRKRPSDNNPFPEPDCSYMGGSNKWLKTKVNEWQVREMSRPTRR; Peak 4: Residues 2–72, SKKIRDFELMSTREICCQLRISSRTLERYRKRPSDNNPFPEPDCSYMGGSNK WLKTKVNEWQVRE MSRPTR; Peak 5: Residues 41–62, PEPDCSYMGGSNKWLKTKVNEW; Peak 6: Residues 9–30, ELMSTREICCQ LRISSRTLERY.

*Gifsy-1 *Xis was digested over a 40-minute period. Three proteolytic fragments were identified by SDS PAGE analysis (Figure 5). Two fragments accumulated in amounts that facilitated N-terminal sequence analysis and matched predicted *Gifsy-1 *Xis peptide products of limited proteolysis with proteinase K. N-terminal sequence analysis verified the identity of the two proteolytic fragments that spanned residues three to 36 and two to 72.

Mass spectrum analysis of *Gifsy-1 *Xis protein digested with proteinase K identified several *Gifsy-1 *Xis peptides that were specifically generated by proteolysis (Figure 4B–4D). These fragments were resistant to further proteolysis by proteinase K. Two additional peptides persisted corresponding to molecular masses of 4.6 kDa (Peak 1 = residues three to 35), and 4.4 kDa (Peak 2 = residues three to 36). Two related peptides, a 8.9 kDa peptide (Peak 3) and a 9.2 kDa peptide (Peak 4), were produced after 20 minutes of proteolysis. The 8.9 kDa peptide was identified as the sequence that spans residues six to 73. This fragment persisted for 160 minutes. The 9.2 kDa fragment represented the sequence that spans residues two to 72 and persisted for 80 minutes in our experimental conditions. The single residue difference observed at the N-terminus of fragments represented by Peaks 1 and 2 and Peak 4 may have been generated by chemical modification, mass spectroscopy-induced fragmentation, or non-specific clipping of the peptides by proteinase K [24,25]. This same phenomenon was observed at the C-terminus of fragments represented by Peaks 3 and 4. We interpret these results to indicate that fragments represented by peaks 1, 2, and 4 likely possess the same proteinase K cleavage site at their N-termini and that fragments represented by peaks 3 and 4 are likely to possess the identical proteinase K cleavage site at their C-terminal sequences. Peaks 5 (2.8 kDa) and 6 (3.1 kDa) were observed after 80 minutes of proteolysis and persisted in proteolysis reactions for 160 minutes. Peak 5 represented the *Gifsy-1 *Xis fragment that spans residues 41 to 62. Peak 6 represents the *Gifsy-1 *Xis fragment that spans the residues nine to 30. Fragments that span the C-terminal tail were not observed during mass spectroscopy analysis of proteinase K digests. This corresponds to our modeling data that suggests that residues 73 to 94 may participate in forming a C-terminal tail that lacks a defined secondary structure.

**Figure 6:**
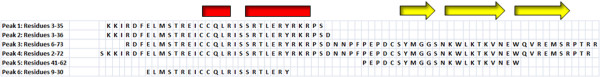
**Alignment of *Gifsy-1 *Xis proteolytic fragments.** Red rectangles indicate the borders of the predicted H-T-H motif and yellow arrows delimit the predicted β-sheet motifs.

Peaks 1 and 2 represent stable proteolysis products and contain the N-terminus of *Gifsy-1 *Xis that includes the putative H-T-H motif (Figure 6). These two fragments span the N-terminus to the putative proline loop at residue 35. This result may indicate that the proline loop represents an exposed motif that is sensitive to proteolysis. Peaks 3 and 4 represent a larger fragment that spans the N-terminus and includes a significant portion of the C-terminal region of the *Gifsy-1 *Xis protein. Peak 5 represents a small fragment that closely corresponds to the predicted *Gifsy-1 *Xis β-sheet. Peak 6 contains a fragment that is predicted to span the N-terminal H-T-H motif. Fragments that are represented by peaks 1, 3, 5, and 6 include both the putative *Gifsy-1 *Xis H-T-H motif and the predicted β-sheet. These fragments persist after 160 minutes of proteolysis with proteinase K and may represent stable motifs within the *Gifsy-1 *Xis protein.

### Mutational Analysis of *Gifsy-1 *Xis

**Figure 7:**
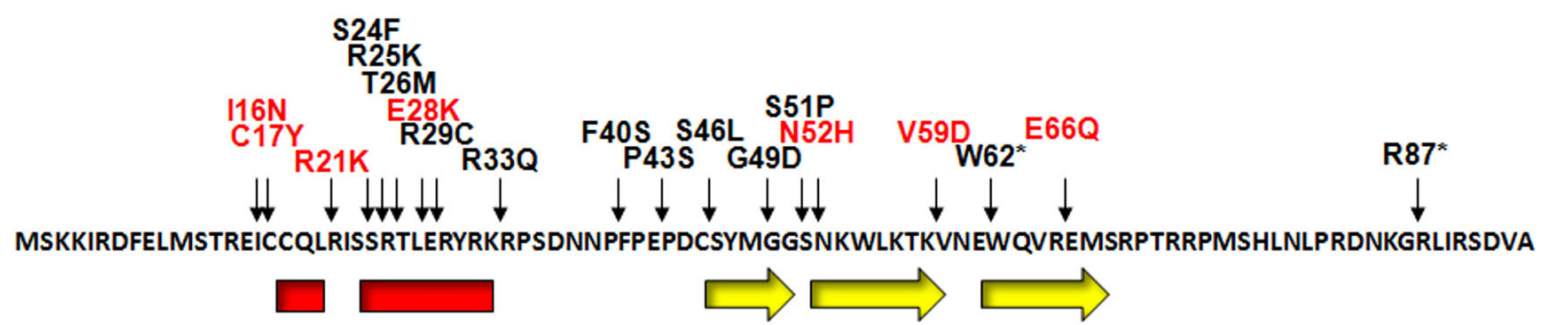
**Position of Gifsy-1 Xis mutants relative to Robetta secondary structure prediction.** Red rectangles indicate sequences with a propensity to form α-helixes. Yellow arrows indicate sequences with a propensity to form β-strands. The positions and identities of random mutations generated in the protein are indicated above the primary amino acid sequence. Red characters indicate mutants generated through mutagenic PCR. Black characters indicate mutations generated through DES mutagenesis.

In order to identify residues that are necessary for the DNA binding activity of the *Gifsy-1 *protein we performed chemical and PCR mutagenesis of the *Gifsy-1 *Xis gene resulting in the isolation of 17 substitution mutants along with two amber mutants. The 19 mutants isolated by PCR and DES chemical methods occurred over almost the entire length of the *Gifsy-1 *Xis protein, though mutations clustered in the two secondary structures predicted by the Robetta modeling program. The N-terminal α-helical region between C18 and K32 contained six of the 17 substitutions. We isolated substitution mutants of four of the six arginine residues in this highly basic region (R21K, R25K, R29C, and R33Q). Six more mutations occurred within the predicted β-sheet between residues S46 and S68. Two mutations occurred within the putative proline loop identified by sequence homology to the several RDF proteins and transcriptional regulators (Figure 7).

**Figure 8:**
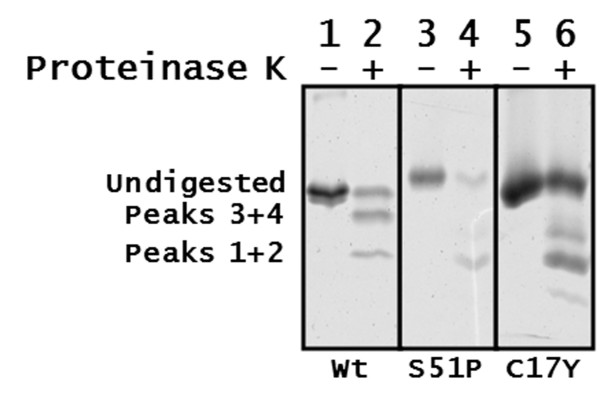
**Limited proteolysis of wild-type *Gifsy-1 *Xis and *Gifsy-1 *Xis substitution mutants S51P and C17Y.** Lanes 1, 3 and 5 contain protein without the addition of proteinase K. Lanes 2, 4, and 6 contain the indicated *Gifsy-1 *wild-type protein or substitution mutants S51P or C17Y. Limited proteolysis was accomplished using a protein to proteinase molar ratio of 80:1. Peaks corresponding to wild-type *Gifsy-1 *mass spectroscopy results are indicated.

The *Gifsy-1 *Xis mutants with the following residue substitutions were purified: Arg29Cys, Ile16Asn, Ser51Pro, Arg25Lys, Cys17Tyr, Phe40Ser, and Ser24Phe. All proteins were tested by EMSA against a radiolabeled DNA probe consisting of the 160 base pairs of the *Gifsy-1 attP *region. The mutant proteins lacked detectable DNA binding activity. To determine the possible structural changes caused by the substitutions, the mutants and the wild-type *Gifsy-1 *Xis were subjected to limited proteolysis. The concentrations of each protein were normalized to that of the wild-type *Gifsy-1 *Xis. Each protein was then subjected to limited proteolysis with proteinase K. A molar ratio of Xis to proteinase of 80:1 was used. Limited proteolysis of wild-type *Gifsy-1 *Xis produced three bands on SDS-PAGE corresponding to undigested protein, peak 1 (residues 3 to 35), and peak three (residues 6 to 73) (Figure 8). Analysis of reactions containing the wild-type protein and proteinase K suggests that peaks 1 and 2 migrate in SDS-PAGE as a single band. Peaks 3 and 4 also migrate together as a single band. With the exception of undigested wild-type *Gifsy-1 *protein, this fragmentation pattern was equivalent to those obtained from proteolytic reactions that utilized a protein to proteinase ratio of 8:1.

Analysis of proteolytic fragments of *Gifsy-1 *Xis wild type protein and substitution mutants indicates that the small stable proteinase K proteolysis fragments that were observed as peaks 1 and 2 during mass spectroscopy analysis were produced from proteolysis of the Ser51Pro and Cys17Tyr substitution mutants. The larger proteolytic fragment represented by peaks 3 and 4 were not apparent in the limited digest of the *Gifsy-1 *Xis Ser51Pro and Cys17Tyr mutants. Limited proteolysis of the Cys17Tyr mutant resulted in the production of a band that represents peaks 1 and 2 and an additional uncharacterized band above 4.6 kDa. These altered peptide fingerprints may be created by changes the 3-dimensional folding pattern of the *Gifsy-1 *Xis by the substitutions at the 17^th ^and 51^st ^residues. The Cys17 residue lies within the predicted N-terminal α-helix and the Ser51 residue lies within the N-terminal β-sheet. This may indicate that the substitutions altered or destabilized the putative interactions of the two predicted motifs. As a result, the relative amount of the small fragment spanning residues 48 to 75 and residues 48 to 94 produced by limited proteolysis with proteinase K increased.

## Conclusion

To further analyze the structure of the *Gifsy-1 *Xis protein, we used Clustal W to perform protein sequence-based homology analysis. We produced a multiple-sequence alignment (MSA) of the *Gifsy-1 *Xis and several predicted and characterized RDF proteins, transcription factors, and AlpA homologs [26]. *Gifsy-1 *Xis aligned with two distinct groups of proteins, (Figures 1 and 2). The proteins in the first group use diverse mechanisms to bind to DNA and RNA, and include the transcription factors, phage RDFs, the AlpA protein, and the multifunctional P4 Vis protein (Figure 1) [27-31]. For example, the *Gifsy-1 *Xis protein is known to bind to a tripartite binding site composed of direct imperfect repeats in the *Gifsy-1 attP *region in a cooperative and sequential manner similar to that of the lambda Xis protein [9]. P4 Vis acts as a lambda Xis-like excisionase protein by binding to DNA in the P4 *attP *region as determined by EMSA and DNase I footprint analysis. The P4 Vis protein also regulates recombination by binding to RNA transcripts [29]. The *Shigella flexineri *Rox protein stimulates Int mediated excision of the *she *pathogenicity island but is not essential for excision [32]. It is not known whether the Rox protein acts exclusively as a lambda-like RDF or as a transcription factor to stimulate excision. These proteins all possess a FPPP loop that follows a predicted N-terminal H-T-H motif. The FPPP loop is preceded by a third region of homology that corresponds to the predicted 3-sheet β-strand of the *Gifsy-1 *Xis protein.

We were led to the characterization of a second group of proteins that share limited homology to the *Gifsy-1 *Xis by the relationship of the *Gifsy-1 *Int and the R391 Int proteins. The *Gifsy-1 *phage encodes an Int with a 36% identity to the Int of the IncJ conjugative element R391 [33]. Clustal W analysis identified a subset RDF proteins belonging to conjugative elements that share homology with the *Gifsy-1 *Xis. In the putative N-terminal motif, the *Gifsy-1 *Xis possesses homology to the Orf2c protein of the CTnDOT conjugative transposon and the R391 Jef protein (Figure 2). The Orf2c protein binds specifically to CTnDOT DNA (Jieyong Lei and A. Salyers, personal communication) and functions in the excision of the CTnDOT element. The R391 Jef protein belongs to the RDFs encoded by a newly characterized SXT/R391 family of mobilizable integrating conjugative elements. All three proteins are predicted to form homologous N-terminal oriented H-T-H motifs. Our structure and sequence analysis of the *Gifsy-1 *Xis protein may add insight into the possible mechanism by which similar proteins such as the AlpA homologs, P4 Vis, Rox, e14 Xis, and the R391 Jef proteins might function.

Three-dimensional folding analysis with Robetta provided results that complemented those we obtained from the sequence-based analysis of the *Gifsy-1 *Xis protein and related DNA binding proteins. Robetta analysis [19] of the *Gifsy-1 *Xis protein indicates that it contains an α-helix rich region near its N-terminus (residues C18 to K32) followed by a β-sheet structure containing three β-strands (residues S46 to S68) (Figure 3A). The predicted α-helix sequence and the β-sheet are connected by a putative proline loop region. The remainder of the protein structure was unresolved by the Robetta tool. A similar structural arrangement was determined by NMR and X-ray analysis for the lambda Xis protein [11,12] (Figure 3D). In the case of the lambda Xis protein, the proline loop positions the third DNA-binding surface of the lambda Xis protein into the minor groove of DNA. In contrast, the NMR structure of the Tn916 Xis protein [34] possesses an arrangement similar to the lambda Xis even though it does not possess a proline loop that connects the helix-rich region to the β-sheet (Figure 3B).

The validation of this *Gifsy-1 *Xis model was investigated using limited proteolysis with proteinase K and random mutagenesis. MALDI-TOF analysis of proteolysis products several fragments of the *Gifsy-1 *Xis protein that are resistant to enzymatic proteolysis. Fragments corresponded to residues three to 36, six to 73, nine to 30, and 41 to 62. The two fragments that include residues nine to 30 and residues 41 to 62 correspond to the predicted H-T-H and β-sheet motifs respectively. Fragments of the C-terminal tail were not observed. This data corresponds to our prediction that the flexible C-terminal region is accessible to proteases and is not expected to possess a rigid globular structure [23,35,36]. Overall, data from proteolytic reactions agreed with the model generated by the Robetta server and suggested that the *Gifsy-1 *Xis might possess a structure similar to that of the lambda Xis and Tn916 Xis proteins.

Random amino acid substitutions generated by DES mutagenesis and mutagenic PCR occurred mostly within the regions of *Gifsy-1 *Xis for which structure predictions were obtained. Mutations of the *Gifsy-1 *Xis protein that occur in these regions (I16N, C17Y, R21K, E28K, N52H, V59D, and E66Q) result in a loss of DNA binding activity. Proteolysis studies of wild-type *Gifsy-1 *Xis and purified Xis substitution mutants suggest that the loss of DNA binding may be the result of altered or unstable protein structure. Our results suggest that residues within predicted helix, proline loop, and β-sheet regions of the *Gifsy-1 *Xis are essential for both proper folding and function of the protein.

Sequence analysis, proteolysis, and mutagenesis suggest that the *Gifsy-1 *Xis possesses at least two surfaces that are involved in DNA binding to *Gifsy-1 attP *DNA. The *Gifsy-1 *Xis protein is required for excision of the *Gifsy-1 *phage and binds to *Gifsy-1 attP *DNA. However, the mechanism through which the *Gifsy-1 *Xis influences the direction of *Gifsy-1 *Int mediated site-specific recombination is still unknown. Our studies suggest that the *Gifsy-1 *Xis protein possesses sequence characteristics of both transcription factors, lambda phage-like RDF proteins, and separately to an uncharacterized group of RDFs belonging to conjugative transposons. It is not known whether the *Gifsy-1 *Xis regulates the excision of the *Gifsy-1 *phage by means other than binding to specific DNA sequences in order to participate in a higher order nucleoprotein complex with *Gifsy-1 *Int, IHF, and *att *DNA.

## Methods

### Chemicals and enzymes

Antibiotics were added to media in the following concentrations: Carbenecillin, 100 μg/mL (Sigma); Kanamycin, 50 μg/mL (Sigma); Chloramphenicol, 20 μg/mL (Sigma). Rifampicin was used at a concentration of 200 μg/mL as previously indicated [9]. Isopropyl-β-D-thiogalactopyranoside (IPTG) was obtained from Research Products International Corp. and was used at a concentration of 1 mM. Diethyl Sulfate (DES) was purchased from Sigma. X-gal was obtained from Research Products International Corp. and was added to media to a concentration of 80 μg/mL. L-arabinose (Sigma) was added to media at a concentration of 1%. Proteinase K was obtained from Invitrogen. Platinum Pfx DNA polymerase (Invitrogen) was used to amplify DNA fragments for cloning into expression vectors. PCR mutagenesis was accomplished using a Genemorph II PCR kit (Stratagene). Automated fluorescent sequencing was carried out at the W. M. Keck Center at the University of Illinois at Urbana Champaign. The restriction enzymes SmaI and BamH1 were used to clone *xis *genes into the pKK223 vector (Fermentas) to result in the creation of the pSM13-1 plasmid. The restriction enzymes NdeI and BamHI were used to clone *xis *genes into the pET27b+ vector (Novagen). T4 DNA ligase used in cloning experiments was obtained from Invitrogen.

### Bacterial strains and plasmids

All strains are derivatives of the *Salmonella *Typhimurium strain MA6052 that was cured of all three *Gifsy *phages (ATCC14028s *Gifsy-1*^-^*, Gifsy-2*^-^*, Gifsy-3*^-^) (Table 1) [37]. MA6052 possesses the wild-type *attB *sequences of all three *Gifsy *phages. This strain was donated by the L. Bossi laboratory. The strain JG15036 (MA6052 *ara702::Tn10dTet*) that contains the wild-type *Gifsy-1 attB *sequence was created by transducing MA6052 with a P22HT lysate of *ara702::Tn10dTet *(*araB*) [37-40]. The disruption of the *araB *gene by *Tn10dTet *allows efficient expression of *Gifsy-1 *Int from the pSM2 vector upon induction with arabinose [41]. Strain JG15049, a derivative of JG15036, contains the Integrase plasmid, pSM2-1 (cam^R^). Strain JG15067, a derivative of JG15049, contains pSM2-1 and the suicide plasmid, pSM3-1 (kan^R^), integrated into the *Salmonella *Typhimurium *Gifsy-1 attB *site. The plasmid pSM3-1 encodes the *oriR6K *origin of replication and will not propagate in a *pir- *strain [42]. *Salmonella *Typhimurium strain JG15073 harbors the pSM13-1 plasmid (amp^R^) and was used in mutagenesis procedures. The strain JG15090 was used to overexpress *Gifsy-1 xis *gene from the pET27b+ vector and to purify wild-type and mutant proteins [9]. The several plasmids used were introduced into bacterial strains via electroporation (2 mm gap cuvette, 400 ohms, 2.4 kV, 25 μF).

**Table 1:** Strain list

**Strains**	**Strain notes**	**Plasmid**	**Integrated element**
MA6052	ATCC14028s; *Gifsy-1-, Gifsy-2-, Gifsy-3-*	N/A	N/A
JG15036	ATCC14028s *ara702*::*Tn10dTet*; *Gifsy-1-, Gifsy-2-, Gifsy-3-*	N/A	N/A
JG15049	ATCC14028s *ara702*::*Tn10dTet*; *Gifsy-1-, Gifsy-2-, Gifsy-3-*	pSM2-1 (*int*)	N/A
JG15067	ATCC14028s *ara702*::*Tn10dTet*; *Gifsy-1-, Gifsy-2-, Gifsy-3-*	pSM2-1 (*int*)	pSM3-1 (*attB*)
JG15073	Expression of *Gifsy-1 *Xis for excision assay	pSM13-1 (*xis*)	N/A
JG15090	Overexpression of *Gifsy-1 *Xis	pET27b+ (*xis*)	N/A

*Gifsy-1 *Xis was encoded on the plasmid pSM13-1 and expressed from a *ptac *promoter [37] on the pET27b+ plasmid. The pSM13-1 plasmid is a derivative of the pKK22-3 plasmid (Fermentas) [43,44]. *Gifsy-1 *Int was encoded on the pBR322 derived plasmid pSM2-1 with expression driven by a *pBAD *promoter [37]. The Pi-dependent plasmid pSM3-1 is a derivative of pRA111 [45] and possesses the wild-type *Gifsy-1 attP *sequence cloned between its BamH1 sites. The pSM3-1 plasmid also encodes a kanamycin resistance cassette and *lacZ *with its promoter and operator sequences.

### Excision Assay

For the integration or excision assay, the plasmids were introduced into a *Salmonella *Typhimurium strain JG15049 (Table 1). This strain was cured of all three *Gifsy *phages and possesses the *attB *sites for the *Gifsy-1*, *-2*, and *-3 *phages. The *Gifsy-1 attP *site was encoded on the *pir*-dependent plasmid pSM3-1. The pSM3-1 plasmid was integrated into the *Gifsy-1 attB *site to form an integrated plasmid flanked by *attL *and *attR *sites after electroporation into strain JG15049 that carries the pSM2-1 plasmid [37]. To achieve integration of the pSM3-1 plasmid, JG15049 cells were grown in Luria Broth (LB) [46] to an O.D. at 600 nm of 0.4. L-arabinose was added (1%) and the cells were further grown to an O.D. 600 of 0.6. Cells were then harvested and made electrocompetent. The pSM3-1 plasmid was then electroporated into the JG15049 strain and cells were recovered in LB broth containing arabinose for 30 minutes and plated onto LB agar containing kanamycin, carbenicillin, and X-gal. Blue, kanamycin resistant colonies were screened by PCR to confirm the integration of the pSM3-1 plasmid into the *Gifsy-1 attB *site (Table 2). The resulting strain that harbors the integrated pSM3-1 plasmid and the pSM2-1 plasmid that encodes *Gifsy-1 *Int under the control of an arabinose inducible promoter was named JG15067.

**Table 2:** Oligo nucleotides.

**Oligonucleotide**	**Sequence**	**Function**
Xis-1060f	TACCCACGCCGAAACAAG	Sequencing of pSM13-1
Xis genemorphf	ATTTCACACAGGAAACAGAATTCCCGGGGGA	PCR mutagenesis
Xis Genemorphr	TCTCATCCGCCAAAACAGAAGCTTGGCTGCAGTTT	PCR mutagenesis
pET27bplusXisNde1f	GCGCGCGCGCCATATGAGCAAAAAAATTAGAGACTTTGAATTGATG	Cloning of *Gifsy-1 xis *into pET27b+
pET27bplusXisBH1r	GCGCGCGCGCGGATCCTCACGCCACGTCAGACCGGATGAGTCGACC	Cloning of *Gifsy-1 xis *into pET27b+
T7 promoter	TAATACGACTCACTATAGGG	Sequencing of pET27b+ constructs

To induce excision, strain JG15067 was induced with 1% L-arabinose and made electrocompetent. The pSM13-1 plasmid, carrying *Gifsy-1 xis*, was then introduced to the cells by electroporation. Cells were recovered in LB broth containing IPTG (0.1 mM) and L-arabinose for one hour. Cultures were then plated onto LB agar containing 1 mM IPTG, 1% L-arabinose, chloramphenicol, and carbenicillin. The plates were incubated at 37°C and grown overnight. Colonies from the plate were then patched onto LB plates containing kanamycin, chloramphenicol and carbenicillin. Colonies that were both carbenicillin and chloramphenicol resistant were scored for the loss of resistance to kanamycin. The loss of kanamycin resistance indicated the expression of functional Xis protein and the excision of the integrated pSM3-1. The retention of kanamycin resistance indicated that a mutation occurred on the pSM13-1 plasmid that resulted in the loss of *xis *expression or a mutation within the *xis *gene that reduced or eliminated the function of the protein.

### Mutagenesis of *Gifsy-1 xis*

Cultures of *Salmonella *Typhimurium strain JG15073 containing the plasmid pSM13-1 were treated with the mutagen DES for 55 minutes to achieve a 90% killing of cells [47]. Cells were then washed to remove DES and were then cultured overnight to amplify the DES exposed pSM13-1 plasmids. Plasmids were harvested from the cells by standard miniprep procedures (Qiagen, Inc.). The plasmids were then electroporated into *Salmonella *Typhimurium excision assay strains JG15067 and expression of *Gifsy-1 int *and *Gifsy-1 xis *genes were induced to identify potential mutants of the *Gifsy-1 xis *gene encoded by the pSM13-1 plasmid. Colonies that retained kanamycin resistance and produce blue color on X-gal plates after 48 hours of incubation were presumed to harbor mutated *Gifsy-1 xis *genes. Plasmids that carried putative *xis *mutations were harvested and back-crossed into the assay strain JG15067 to confirm the results of the excision screen. Individual mutant plasmids were isolated and all mutants were sequenced using primers that annealed to the pSM13-1 plasmid (Table 2).

In order to eliminate the occurrence of mutations outside of the *xis *gene, an in vitro PCR mutagenesis technique was also used. Mutagenic PCR was carried out using a Stratagene Genemorph II random mutagenesis kit. Briefly, primers (Integrated DNA Technologies) were designed to anneal to sequences immediately 5' and 3' to the *Gifsy-1 xis *gene on the plasmid pSM13-1 (Table 2). Mutagenic PCR reactions that contained 100 ng of the initial *Gifsy-1 *Xis target DNA were allowed to run for 20 cycles in order to generate an average of 4.5 mutations per 1 kb of DNA. The total PCR reaction was then digested with restriction enzymes and cloned in-frame with the *ptac *promoter in the pKK22-3 plasmid. The PCR inserts were then ligated into the pKK22-3 plasmid and electroporated into *Salmonella *Typhimurium strain 14028s carrying the pSM2-1 plasmid and integrated pSM3-1 plasmid. Putative mutants were backcrossed and sequenced as described above.

### Protein purification

Purification of the wild-type and mutant *Gifsy-1 *Xis proteins was carried out as previously described [9]. Exceptions were made for amber mutations that resulted in truncations of *Gifsy-1 *Xis with molecular masses of less than 10 kDa. For these truncation mutants, Millipore Centrifugal concentrators with a molecular mass cut off of five kDa were used instead of concentrators with a molecular mass cut off of 10 kDa.

### EMSA

Gel shift analysis of the binding of *Gifsy-1 *Xis and *Gifsy-1 *Xis substitution mutants was accomplished as previously described [9]. Briefly, the *attP*306 DNA fragment (a PCR product containing the *Gifsy-1 attP *sequence) was used in binding reactions at a concentration of 0.5 nM. Proteins were incubated with the DNA ligand for 45 minutes and then loaded onto and resolved on 8% native polyacrylamide gels.

### Limited Proteolysis

*Gifsy-1 *Xis peptides were generated by digesting aliquots of protein in purification buffer (50 mM NaCl, 50 mM HEPES pH 7.0, 10 mM EDTA pH 8.0, 5% Glycerol, 1 mM DTT) with proteinase K. To determine the time course of proteolysis, 10 μg of *Gifsy-1 *Xis was diluted into 200 μL of purification buffer. A volume of 14 μL was removed and immediately denatured in SDS loading buffer (Tris Base pH 8.3 100 mM, Tricine 100 mM, SDS 0.1%) before the addition of proteinase K. This procedure was repeated for every time point after the addition of proteinase K. The final protein to proteinase K ratio was 8:1 (molar ratio) and proteolysis was carried out at room temperature. Aliquots were loaded onto 16% tricine-SDS-PAGE gels (49.5% T, 6% C mixture), resolved, and stained with Sypro Ruby (Invitrogen) [48]. For analysis of mutants, a protein to proteinase K ratio of 80:1 was used. For the preparation of peptides for N-terminal sequence analysis and MALDI-TOF analysis, 20 pmol of *Gifsy-1 *Xis were digested using a 80:1 (protein to proteinase) molar ratio of proteinase K. N-terminal sequence analysis and MALDI-TOF of peptides from wild-type *Gifsy-1 *Xis were carried out by the Protein Sciences Facility at the University of Illinois at Urbana-Champaign. Ten μg of purified *Gifsy-1 *Xis substitution mutants were dissolved in 50 μL of purification buffer. Each solution was then diluted to 200 μL in preparation for proteolysis with proteinase K. Proteolysis reactions were allowed to run for 10 minutes with an 80:1 protein to proteinase K ratio and resolved by Tricine-SDS-PAGE.

### Spectral Analysis and Peak Assignment

Protein and peptide samples were submitted to the Proteomics laboratory at the University of Illinois. Samples were resolved on a MALDI-TOF apparatus. Mass data was then analyzed using the mMass software package [49].

## Authors' contributions

AF contributed to the design of the study, carried out all studies, and drafted the manuscript. JG conceived of the study, contributed to its design and coordination, and edited the manuscript in preparation for publication.
